# Synergistic Antibacterial Activity of an Active Compound Derived from *Sedum takesimense* against Methicillin-Resistant *Staphylococcus aureus* and Its Clinical Isolates

**DOI:** 10.4014/jmb.2105.05015

**Published:** 2021-07-21

**Authors:** Eun-Tak Jeong, Seul-Ki Park, Du-Min Jo, Fazlurrahman Khan, Tae Ho Choi, Tae-Mi Yoon, Young-Mog Kim

**Affiliations:** 1 DYNE SOZE Co., Ltd., U-TOWER, Yongin 16827, Republic of Korea; 2 Department of Food Science and Technology, Pukyong National University, Busan 48513, Republic of Korea; 3 Institute of Food Science, Pukyong National University, Busan 48513, Republic of Korea; 4 Research Center for Marine Integrated Bionics Technology, Pukyong National University, Busan 48513, Republic of Korea

**Keywords:** Antibiotic resistance, clinical isolates, methicillin-resistant, *Sedum takesimense*, *Staphylococcus aureus*, synergy

## Abstract

There are a growing number of reports of hospital-acquired infections caused by pathogenic bacteria, especially methicillin-resistant *Staphylococcus aureus* (MRSA). Many plant products are now being used as a natural means of exploring antimicrobial agents against different types of human pathogenic bacteria. In this research, we sought to isolate and identify an active molecule from *Sedum takesimense* that has possible antibacterial activity against various clinical isolates of MRSA. NMR analysis revealed that the structure of the HPLC-purified compound was 1,2,4,6-tetra-O-galloyl-glucose. The minimum inhibitory concentration (MIC) of different extract fractions against numerous pathogenic bacteria was determined, and the actively purified compound has potent antibacterial activity against multidrug-resistant pathogenic bacteria, *i.e.*, MRSA and its clinical isolates. In addition, the combination of the active compound and β-lactam antibiotics (*e.g.*, oxacillin) demonstrated synergistic action against MRSA, with a fractional inhibitory concentration (FIC) index of 0.281. The current research revealed an alternative approach to combating pathogenesis caused by multi-drug resistant bacteria using plant materials. Furthermore, using a combination approach in which the active plant-derived compound is combined with antibiotics has proved to be a successful way of destroying pathogens synergistically.

## Introduction

Various antibiotics have been used to treat microbiological infections or diseases; however, several organizations, including the CDC (Centers for Disease Control and Prevention) and the U.S. Food and Drug Administration (U.S. FDA) have issued repeated warnings regarding their overuse [1]. Furthermore, antibiotic overuse is a growing concern among global public health authorities, and carries with it the significant risk of propagating antibiotic-resistant bacteria. According to CDC reports, at least 2 million people become infected each year, and 23,000 people die in the United States as a result of infection with multi-drug resistant pathogenic bacteria [2]. Every year, the number of people infected by antibiotic-resistant bacteria rises, particularly methicillin-resistant *Staphylococcus aureus* (MRSA), which poses a severe threat to human health [3]. MRSA is resistant to antibiotics with methicillin groups, with the exception of glycopeptides such as vancomycin and teicoplanin [4]. Thus, overuse of vancomycin to treat MRSA infection resulted in the development of vancomycin-resistant *S. aureus* (VRSA) [5]. Furthermore, numerous studies have shown that not only the type strain of MRSA, but also several clinically isolated MRSA, exhibit antibiotic resistance [6]. As a result, various alternative strategies for controlling infections caused by antibiotic-resistant bacteria have been developed, including the use of natural or synthetic medicines, as well as the combination of these treatments with conventional antibiotics to produce synergistic antimicrobial effects [7, 8].

Many studies on the antibacterial effects of phytochemicals derived from plants as natural therapies have been published [9]. These plant-derived antibacterial compounds have also been widely used in combination therapies in conjunction with conventional antibiotics [10-13]. Choi *et al*. reported that eckol, a compound derived from *Ecklonia cava*, has a bactericidal impact on MRSA and has synergistic bactericidal action when combined with β-lactam group antibiotics [10]. Similarly, plant extracts from *Camellia sinensis*, *Delonix regia*, and *Holarhena antidysenterica* have been shown in studies to have a bactericidal effect on MRSA, methicillin-susceptible *S. aureus* (MSSA), and clinical isolates. [11, 12]. Furthermore, when combined with antibiotics such as tetracycline, chloramphenicol, and ampicillin, these plant extracts have synergistic bactericidal properties. [11, 12]. Recently, it was revealed that a cryptotanshinone compound isolated and purified from *Salvia miltiorrhiza* had antibacterial activity against MRSA, VRSA, and MSSA [13]. When combined with β-lactam antibiotics such as oxacillin, ampicillin, and vancomycin, this pure compound also exhibited a synergistic effect [13].

Here, we achieved our goal of isolating and purifying a bioactive component from *S. takesimense* capable of efficiently killing MRSA and its clinical isolates. Furthermore, combination therapies using isolated molecules and β-lactam antibiotics were used to kill MRSA and its clinical isolates in a synergistic manner. The present study provides new insight on the use of a plant-derived natural product as an alternative strategy for killing multi-drug resistant bacterial infections..

## Material and Methods

### Extraction of Plant Material

The aerial part of *S. takesimense* was purchased from Arim SG Co., Ltd. botanical garden in Gyeongbuk Province, the Republic of Korea, in July 2019, and dried at 80°C for 4 days using a dry oven. The dried *S. takesimense* was rigorously extracted with a 10-time volume of 94% ethanol at room temperature for 30 h. The yield of the extracted and concentrated compound was found to be 15% (w/w). The extract was stored at -20°C before purification and future experiment in the anti-MRSA test.

### Purification and Characterization of Active Compounds

The crude extract of powdered *S. takesimense* (20 g) was subjected to a silica gel column (3.5 by 60.0 cm; Kiesel gel 60, 150 g, 230 to 400 mesh; E. Merck, Darmstadt, Germany) with an eluting solvent of chloroform/methanol/distilled water/acetic acid (55:36:8:1 v/v) to get first fractions. After the first fraction, 4 fractions were successfully separated using a Sephadex LH20 column (3.5 by 60 cm; 130 g, 70 to 100 μm; Sigma-Aldrich, USA) in the presence of methanol as described earlier [14, 15]. These 4 fractions were further purified using a preparative high-performance liquid chromatographic (prep-HPLC) system (Capcell Pak C18, 20 by 250 mm; Shiseido, Japan). The substances were eluted with an isocratic solvent system such as methanol/water (20:80 v/v) at a flow rate of 15.0 ml/min and detected at a wavelength of 270 nm using a Waters 996 PDA detector (Waters, USA). With the help of prep-HPLC systems, a purified compound can be obtained.

The chemical structure of the purified compound was determined using proton nuclear magnetic resonance (^1^H-NMR) spectroscopy. The pure compound was also identified using a mass spectroscopy MSD1100 single quadrupole equipped with an ESI (Electrospray ionization, Hewlett-Packard Co., USA). ^1^H-NMR spectra were recorded by dissolving the pure compound in the deuterated methanol (CD3OD; Merck) and analyzed using a Bruker AMX-500 (Bruker Analytische Messtechnik GmbH) spectrometer, which was operated at 500 MHz for 1 h. The chemical shifts (parts per million) are pertinent to tetramethylsilane, which was used as an internal standard [15]. Coupling constants are reported in hertz, and the chemical structure of the purified single compound was analyzed based on the ESI-MS and ^1^H-NMR spectral data and comparisons of previously reported values [16-24].

### Bacterial Strains and Antibiotics

The reference pathogenic bacteria used in the present study for the antimicrobial screening were purchased from the Korean Collection for Type Culture (KCTC; Korea) and included *Escherichia coli* (EC, KCTC 1682), *Pseudomonas aeruginosa* (PA, KCTC 4562), *Salmonella* Typhimurium (ST, KCTC 1925), *Vibrio parahaemolyticus* (VP, NCTC 11344), *Bacillus cereus* (BC, KCTC 3624), *Listeria monocytogenes* (LM, KCTC 3569), *Staphylococcus aureus* (SA, KCTC 1927), *Staphylococcus epidermidis* (SE, KCTC 3958), and *Cutibacterium acnes* (CA, KCTC 3314). The standard bacterial strain of methicillin-resistant *Staphylococcus aureus* (MRSA, KCCM 40511) used in this study was purchased from the Korean Culture Center of Microorganisms (KCCM; Korea). The seven clinical MRSA isolates were generously provided by Dong-A University Hospital (Korea). All bacterial strains were aerobically cultivated at 37°C in Mueller-Hinton broth (MHB; Difco, USA) or tryptic soy broth (TSB; Difco) growth media. All antibiotics and standard compounds used in this study were of analytical grade and purchased from Sigma (Sigma-Aldrich, USA).

### Determination of Antibacterial Activity and MICs of the Extracts and Purified Compound

The antimicrobial activity of the crude extracts from *S. takesimense* was firstly screened based on the appearance of the inhibitory zone using the agar diffusion method as described by Dickson *et al*. [25] with a slight modification. The zone of inhibition that appeared on the tryptic soy agar (TSA) plate after 24 h of incubation was recorded. In one well, DMSO was also loaded as a negative control, and its value was subtracted from the test sample. The MICs were determined using a two-fold serial dilution method as recommended by the National Committee for Clinical Laboratory Standards (USA) [26].

### Synergistic Effects of Active Compounds in Combination with Antibiotics Against MRSA

The interaction between active components present in the extract or purified compounds, with conventional antibiotics such as ampicillin, oxacillin, and clavulanic acid against MRSA, was tested as recommended by the checkerboard method [27]. The synergistic effect was evaluated by summing up the FICs to calculate the FIC index, which indicated synergy when the index values were: ≤ 0.5, synergic; > 0.5 to ≤ 1, additive; > 1 to ≤ 2, independent; > 2, antagonistic [28].

## Results and Discussion

### Purification and Structural Determination of the Compound

The ethanolic extract of *S. takesimense* was purified using chromatography strategies with silica gel columns, Sephadex LH-20 columns, and a prep-HPLC analytical system. As described in Materials and Methods, several strategies were tried to isolate and purify a single compound from *S. takesimense*. In the crude extract using ethanol, the extraction yield was 15% (w/w), and 30 g of extract was obtained from 200 g of raw material. Afterwards, 1.4 g of extract was obtained using Sephadex LH 20 column chromatography. Then, using the 1.4 g of extract, 3 fractions were obtained using the prep HPLC (fractions 4, 5, and 6), and among them, TOGG was isolated and purified from fraction 5 (63.2 mg). The HPLC chromatogram of the compound TOGG with retention time 15.2 min from fraction 5 is provided in the supplementary material (Fig. S1). The chemical structure of the purified compound was determined with the help of proton nuclear magnetic resonance (^1^H-NMR) spectroscopy. The NMR spectrum of the compound is given in the supporting information (Fig. S2). The chemical shifts of hydroxyl proton in galloyl and glucose in the TOGG are also summarized in Table S1.

**Fig. 1:**
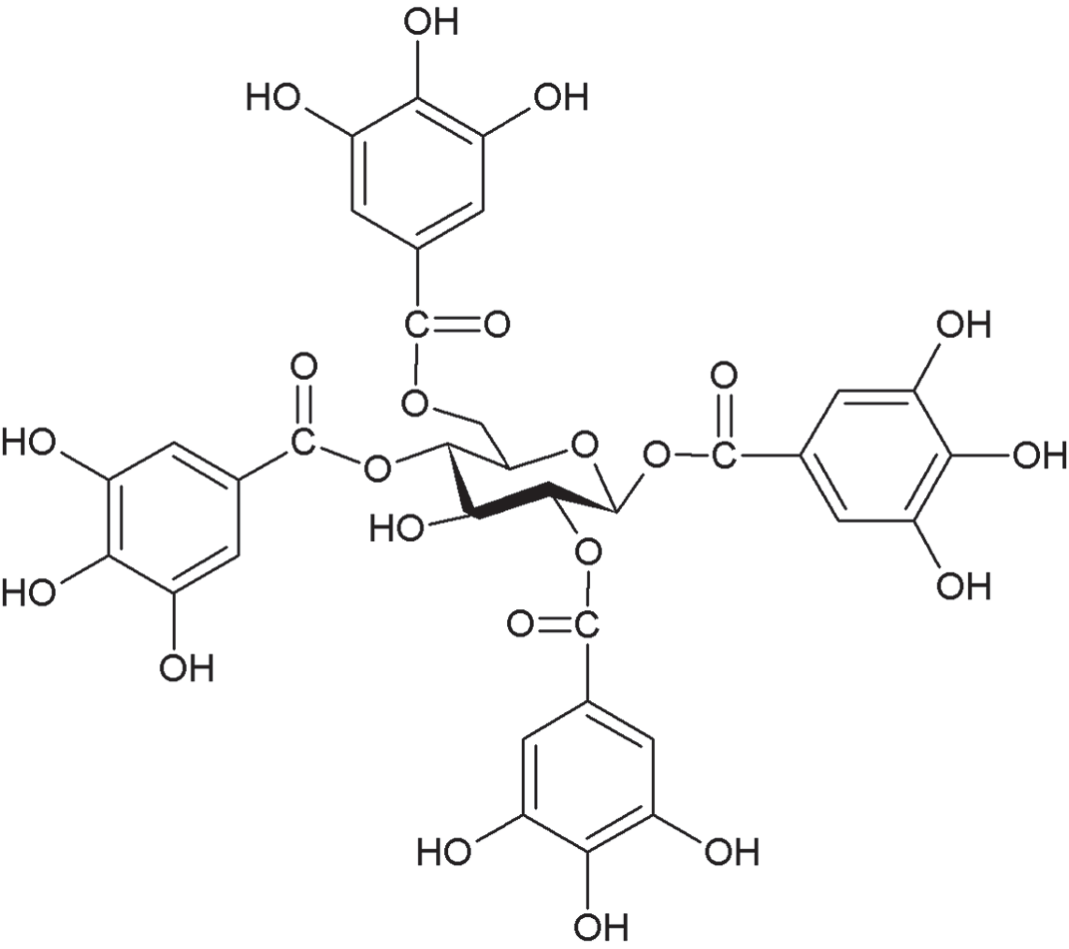
Chemical structure of purified compound (1,2,4,6-tetra-O-galloyl-β-glucose) identified from aerial parts of *Sedum takesimense*.

Furthermore, the obtained spectra have also been compared with the previously reported spectra [29]. Hence, based on the spectroscopic studies, the isolated compound was identified as 1,2,4,6-tetra-O-galloyl-β-glucose (TOGG). Fig. 1 shows the chemical structure of pure compounds isolated from aerial parts of *S. takesimense*. Two standard compounds, penta-galloyl-glucose (PGG) and pyrogallol (PG), were also selected to evaluate the anti-MRSA activity, and the obtained results were compared with the identified compound (TOGG). Furthermore, PGG and PG were also used to evaluate the synergistic effect in combination with β-lactam antibiotics towards MRSA and its clinical isolates.

### Screening of Antibacterial Activity of Crude Extracts and Purified Compound towards Different Pathogenic Bacteria

The antibacterial activity of the crude extract was first tested using the agar diffusion method, and the obtained MIC results are shown in Table 1. These results indicate that the crude extract of *S. takesimense* at two different concentrations (1 mg and 5 mg) has bactericidal activity against nine tested pathogenic bacteria. The zones of inhibition in diameter (mm) towards different bacterial strains were found in the order of SE > VP > ST > PA = BC > EC > CA > SA > LM, respectively. Meanwhile, towards gram-positive bacteria such as SA and LM, a lower inhibition zone was recorded as compared to the other pathogenic bacteria. The representative agar plate showing the zone of inhibition for each bacterium is also provided in the supporting information (Fig. S3). Also, among the gram-negative bacteria, CA had the lowest inhibition zone. These MIC results are in close agreement with the previous result of the antibacterial activity of *S. hybridum* extract [30]. According to Odontuya *et al*. [30], the prepared crude extract from *S. hybridum* has been reported to exhibit the lowest inhibition zone against the CA.

MIC screening for antibacterial activity was carried out using crude extracts (CE), fractions 4, 5, 6 (F4, F5, F6), and purified compound (TOGG) towards different pathogenic bacterial species as shown in Table 1. Among them, F5 showed the highest effect on the inhibition of pathogenic bacteria growth. The MIC value of the F5 fraction required to inhibit pathogenic bacterial growth was found to be 256 μg/ml for EC, 128 μg/ml for PA and ST, 32 μg/ml for BC and LM, and 16 μg/ml for SA and SE. The pure compound (TOGG) showed the highest inhibitory activity at lower concentrations as compared to other fractions. It required a concentration of 32 μg/ml to inhibit EC and BC growth, 128 μg/ml against PA, ST, and LM, and 16 μg/ml against SA and SE. The purified compound (TOGG) showed higher activity in inhibiting the growth of gram-positive bacteria such as *Staphylococcus* species. The obtained results are in close agreement with the results reported by several previous studies [30-33]. These studies reported that a purified compound extracted from plants effectively inhibits gram-positive bacteria. Therefore, the inhibitory activity against MRSA and its clinical isolates, as well as the synergistic effect with existing antibiotics, were examined using crude extracts, different fractions (F4, F5, and F6), and purified compound (TOGG).

### Bactericidal Effect of Active Compound Against MRSA and its Clinical Isolates

The results of the MIC values against MRSA and its clinical isolates were shown in Table 2. The results showed that fractions 1, 2 and 3 had no bactericidal effect on these pathogens (data not shown). The different extracts derived from *S. takesimense*, such as crude extract, F4, F5, F6, and purified TOGG, and standard compounds (PGG, and PG), inhibited cell growth of all bacterial strains. The inhibitory activity of these extracts and pure compounds was found to differ according to the type of sample or fractions. Among all of the tested compounds, TOGG showed the most potent in vitro antibacterial activity against MRSA and its clinical isolates with MIC values ranging from 16 to 256 μg/ml. In particular, the MIC value of TOGG towards standard strain and clinically isolated strains 6, 8, 10, and 12 appeared to be 16 μg/ml, whereas the MIC values of TOGG against clinically isolated strains 13 and 18 were found to be 32 μg/ml. This singly purified compound derived from *S. takesimense* exhibits a higher inhibitory effect on bacterial cell growth as compared to the standard compounds such as PGG and PG. Several other studies have reported that extracts prepared from natural sources such as plants or seaweeds have high efficacy of antibacterial activity [31, 32]. According to Bensouici *et al*. [31], compounds isolated from Sedum caeruleum have antibacterial activities against clinically isolated pathogenic bacteria such as *E. coli*, *P. aeruginosa*, *Klebsiella pneumoniae*, *S. aureus*, respectively. Also, Lee *et al*. [32] reported a synergistic antibacterial effect of dieckol isolated from *Ecklonia stolonifera* algae in combination with β-lactam-type antibiotics against MRSA. Hence, based on previous and present studies, it was concluded that compounds isolated from plant materials exhibit potent antibacterial activities and show a synergy when applied in combination with β-lactam antibiotics. Therefore, the present study was carried out to determine the anti-MRSA activity of compounds derived from *S. takesimense* towards both clinical and non-clinical isolates and also to investigate the synergistic effect in combination with β-lactam antibiotics.

### MIC of β-Lactam Antibiotics Against Clinically Isolated MRSA

MRSA and all its clinical isolates were found to be highly resistant to the β-lactam antibiotics, including ampicillin and oxacillin, as evidenced from the MIC values where they were found to be either equal to or greater than 64 μg/ml (Table 3). However, in clavulanic acid, the MIC values were either equal to or less than 64 μg/mL towards the MRSA standard strain and its 7 clinical isolates. Several studies have reported that the clinical isolates of MRSA exhibit a varied range of MIC values [33-35]. Other previous studies also reported that clavulanic acid has a bactericidal effect towards MRSA, and especially, that it is more effective when applied in combination with other antibiotics such as amoxicillin [34, 35]. The present study showed a similar bactericidal effect of β-lactam antibiotics such as ampicillin, oxacillin, and clavulanic acid towards MRSA and its clinical isolates.

The MIC values of TOGG compound that were isolated from *S. takesimense* were found to be in the range of 16-32 μg/ml towards MRSA and all clinical isolates, except for isolate No. 14 (Table 2). The MIC values of pure compound TOGG were less than those of the β-lactam- class antibiotics against MRSA and its clinical isolates. The MIC determination result for the antibiotics showed that the MRSA isolate No. 12 was highly sensitive to clavulanic acid as compared to other antibiotics such as ampicillin and oxacillin (Table 3). Similar results have also been found for MRSA KCCM 40511, and MRSA isolate No. 6. Among β-lactam antibiotics, ampicillin was the least effective against MRSA and its isolates, followed by oxacillin, whereas clavulanic acid inhibits the growth of MRSA and its isolates most effectively.

### Synergistic Effects of Active Compound in Combination with β-Lactam Antibiotics Against MRSA

The present study determined the synergistic effects of an active compound derived from *S. takesimense* towards MRSA and several of its clinical isolates in combination with commercial β-lactam antibiotics such as ampicillin, oxacillin, and clavulanic acid. As shown in Table 4, the MIC value of crude extract combined with oxacillin against MRSA strain KCCM 40511 was reduced dramatically from 256 to 0.25 μg/ml. Similarly, the MIC values of F5 and TOGG in combination with oxacillin reduced from 16 to 4 μg/ml and 6 to 2 μg/ml, respectively. The FIC indices of crude extract, F5, and TOGG, when combined with ampicillin, were the same value, *i.e.*, > 1.0. However, the FIC values of the TOGG compound, when combined with oxacillin, were found to be in the range of 0.281 to 0.562 (Table 4). The FIC value of TOGG in combination with oxacillin was found to be 0.281, which exhibited a high effect on MRSA. Fig. S4 shows a representative 96-well microtiter plate used for the checkerboard assays of crude extract (CE), fraction No. 5 (F5), and single compound (TOGG) with ampicillin antibiotic against methicillin-resistant *S. aureus* KCCM 4051. These findings suggest that the combination of TOGG and oxacillin synergistically inhibits MRSA cell growth. Previous reports showed varied synergistic inhibition of MRSA cell growth by β-lactam in combination with epigallocatechin gallate (EGCg) and dieckol [32, 33].

## Conclusion

In this study, we isolated an active compound from the plant species *S. takesimense* utilizing solvent extraction procedures and chromatographic techniques. NMR analysis was also used to analyze the isolated component. The bioactive molecule identified from *S. takesimense* has been given the name 1,2,4,6-tetra-O-galloyl-glucose (TOGG). The isolated compound TOGG was found to show effective bactericidal activity towards MRSA and several of its clinical isolates. In addition, this compound also potentiates the efficacy of conventional antibiotics when applied in combination, and the results were found to be a synergistic way of killing MRSA and its clinical isolates. With the increase in vancomycin prescription, the sensitivity of MRSA to vancomycin has decreased in recent years [32]. Therefore, there is also a need to discover new drugs and alternative therapies to combat vancomycin-resistant microbial pathogens. The present study provides new insight for applying plant-derived bioactive compounds to effectively combat the pathogenesis caused by bacteria, especially MRSA. Future study is required to explore the molecular mechanism involved in killing these pathogenic bacteria by the newly isolated TOGG. Further investigation is also needed to address the mechanism of synergistic killing of this compound when combined with conventional antibiotics.

## Supplemental Materials

Supplementary data for this paper are available on-line only at http://jmb.or.kr.

## Figures and Tables

**Table 1:** Antibacterial activity and MIC values of crude extract, its fraction, and pure compound from *S. takesimense*.

Strain	Antimicrobial activity of 40% ethanolic extract						

	Zone of inhibition (mm)		MIC (μg/mL)				
	
	CE		CE	Frc. 4	Frc. 5	Frc. 6	TOGG

	1mg	5mg					
*Escherichia coli* KCTC 1682	-	9.0±0.5	1024	1024	256	256	32
*Pseudomonas aeruginosa* KCTC 4562	-	9.7±0.5	1024	512	128	512	128
*Salmonella typhimurium* KCTC 1925	-	11.3±0.5	1024	1024	128	512	128
*Vibrio parahaemolyticus* NCTC 11344	-	11.7±0.5	-	1024	-	-	-
*Bacillus cereus* KCTC 3624	-	9.7±0.5	512	512	32	64	32
*Listeria monocytogenes* KCTC 3569	-	2.7±0.8	512	64	32	64	128
*Staphylococcus aureus* KCTC 1927	-	7.3±0.5	512	256	16	256	16
*Staphylococcus epidermidis* KCTC 3958	8.3±0.5	15±0.8	256	64	16	64	16
*Cutibacterium acnes* KCTC 3314	-	7.7±0.5	-	-	-	-	-

*CE, Crude extract of *S.takesimense* with 40% Ethanol; F4, fraction 4; F5, fraction 5; F6, fraction 6; TOGG, and single compound (1,2,4,6-tetra-O-galloyl-β-glucose).

**Table 2:** Minimum inhibitory concentration (MIC) values of crude extracts, fractions, standard compounds, and singly purified compound against methicillin-resistant *Staphylococcus aureus*.

Strain	Source or Reference	Minimum inhibitory concentration (μg/mL) *						

		CE	F4	F5	F6	SC	PGG	PG
KCCM 40511	Standard strain	256	64	16	32	16	128	128
Isolated 6	Clinical isolate**	1024	1024	64	64	16	64	64
Isolated 8		1024	1024	64	64	16	64	128
Isolated10		1024	1024	32	64	16	64	128
Isolated12		512	32	16	64	16	128	64
Isolated13		1024	512	16, 32	64	32	64	256
Isolated14		1024	512	512	64	256	64	128
Isolated18		1024	1024	32	64	32	64	512

*CE, Crude extract of *S. takesimense* with 40% Ethanol; F4, fraction 4; F5, fraction 5; F6, fraction 6; SC, single compound; PGG, penta-galloyl-glucose(standard compound 1); PG, pyrogallol (standard compound 2).

**MRSA KCCM 40511 is a standard strain purchased from KCCM; isolated strains were provided by Dong-A University Hospital.

**Table 3:** Minimum inhibitory concentration (MIC) values of antibiotics (ampicillin, oxacillin, and clavulanic acid) against methicillin-resistant *Staphylococcus aureus* and its clinical isolates.

Strain^*^	Minimum inhibitory concentration (μg/mL)		

	Ampicillin	Oxacillin	Clavulanic acid
KCCM 40511	256	128	32
Isolated 6	128	128	32
Isolated 8	256	64	64
Isolated10	128	64	64
Isolated12	128	256	8
Isolated13	64	64	16
Isolated14	64	64	16
Isolated18	128	64	32

*MRSA KCCM 40511 is a standard strain purchased from KCCM; isolated strains were provided by Dong-A University Hospital.

**Table 4:** Fractional inhibitory concentration (FIC) indexes (FICIs) of combined effects among major active compounds (crude extract, fraction 5, and pure compound), standard compounds (Penta-galloyl-glucose and pyrogallol), and antibiotics against methicillin-resistant *Staphylococcus aureus* KCCM 40511.

Strain	Antibiotics	Compound**	MIC (μg/mL)		∑FIC_max_^a)^	∑FIC_min_^b)^	FICI^c)^

			Alone	Combination			
MRSA KCCM 40511*	Ampicillin	CE	256	32	2.250	0.625	1.031
		AMP	256	128			
		F5	16	0.016	1.250	0.750	1.031
		AMP	256	256			
		SC	16	2	1.250	0.625	1.031
		AMP	256	256			
		PGG	128	64	1.063	0.625	0.844
		AMP	256	128			
		PG	128	2	1.000	0.125	0.262
		AMP	256	64			
	Oxacillin	CE	256	0.25	1.500	0.501	0.531
		OXA	128	64			
		F5	16	4	0.625	0.500	0.562
		OXA	128	32			
		SC	16	2	0.313	0.188	0.281
		OXA	128	8			
		PGG	128	1	0.125	0.064	0.070
		OXA	128	8			
		PG	128	4	2.000	0.250	0.773
		OXA	128	64			
	Clavulanic acid	CE	256	256	2.000	2.000	2.000
		CLA	32	32			
		F5	16	8	2.000	1.500	1.750
		CLA	32	32			
		SC	16	8	2.000	1.500	1.750
		CLA	32	32			
		PGG	128	32	1.125	0.281	0.434
		CLA	32	8			
		PG	128	-	-	-	-
		CLA	32	-			

^a)^ΣFIC_max_, maximum FIC value; ^b)^ΣFIC_min_, minimum FIC value; ^c)^FICI, The FIC index indicated synergistic effect: <0.5, marked synergy; 0.5 to <1.0, weak synergy; 1.0, additive; >1.0 to <2.0, subadditive; 2.0, indifferent; >2.0, antagonistic.

*MRSA KCCM 40511 is a standard strain purchased from KCCM; isolated strains were provided by Dong-A University Hospital. **CE, Crude extract with 40% Ethanol; F4, fraction 4; F5, fraction 5; F6, fraction 6; SC, single compound; PGG, pentagalloyl-glucose (standard compound 1); PG, pyrogallol (standard compound 2).

## References

[1] Ventola CL (2015). The antibiotic resistance crisis: part 1: causes and threats. Pharm. Ther..

[2] Center for Disease Control (CDC) Antibiotic Resistance Threats in the United States.

[3] Tong SY, Davis JS, Eichenberger E, Holland TL, Fowler VG (2015). *Staphylococcus aureus* infections: epidemiology, pathophysiology, clinical manifestations, and management. Clin. Microbiol. Rev..

[4] Kaur DC, Chate SS (2015). Study of antibiotic resistance pattern in methicillin resistant *Staphylococcus aureus* with special reference to newer antibiotic. J. Glob. Infect. Dis..

[5] French GL (2010). The continuing crisis in antibiotic resistance. Int. J. Antimicrob. Agents.

[6] Pai V, Rao VI, Rao SP (2010). Prevalence and antimicrobial susceptibility pattern of methicillin-resistant *Staphylococcus aureus* [MRSA] isolates at a tertiary care hospital in Mangalore, South India. J. Lab. Physicians.

[7] Okwu MU, Olley M, Akpoka AO, Izevbuwa OE (2019). Methicillin-resistant *Staphylococcus aureus* (MRSA) and anti-MRSA activities of extracts of some medicinal plants: a brief review. AIMS Microbiol..

[8] Compean KL, Ynalvez RA (2014). Antimicrobial activity of plant secondary metabolites: a review. Res. J. Med. Plant..

[9] Sato M, Tanaka H, Yamaguchi R, Kato K, Etoh H (2004). Synergistic effects of mupirocin and an isoflavanone isolated from *Erythrina variegata* on growth and recovery of methicillin-resistant *Staphylococcus aureus*. Int. J. Antimicrob. Agents.

[10] Choi JG, Kang OH, Brice OO, Lee YS, Chae HS, Oh YC (2010). Antibacterial activity of *Ecklonia cava* against methicillinresistant *Staphylococcus aureus* and *Salmonella* spp. Foodborne Pathog. Dis..

[11] Aqil F, Khan MSA, Owais M, Ahmad I (2005). Effect of certain bioactive plant extracts on clinical isolates of β‐lactamase producing methicillin resistant *Staphylococcus aureus*. J. Basic. Microbiol..

[12] Aqil F, Ahmad I, Owais M (2006). Evaluation of anti‐methicillin‐resistant *Staphylococcus aureus* (MRSA) activity and synergy of some bioactive plant extracts. Biotechnol. J..

[13] Cha JD, Lee JH, Choi KM, Choi SM, Park JH (2014). Synergistic effect between cryptotanshinone and antibiotics against clinic methicillin and vancomycin-resistant *Staphylococcus aureus*. Evid. Based Complement. Alternat. Med..

[14] Yoon MY, Choi GJ, Choi YH, Jang KS, Park MS, Cha B (2010). Effect of polyacetylenic acids from *Prunella vulgaris* on various plant pathogens. Lett. Appl. Microbiol..

[15] Yoon MY, Choi NH, Min BS, Choi GJ, Choi YH, Jang KS (2011). Potent in vivo antifungal activity against powdery mildews of pregnane glycosides from the roots of *Cynanchum wilfordii*. J. Agric. Food. Chem..

[16] Gangadhar M, Bhavana P, Sunil Y, Datta S (2011). Isolation and characterisation of gallic acid from *Terminalia bellerica* and its effect on carbohydrate regulatory system in vitro. Int. J. Res. Ayurveda. Pharm..

[17] Hisham DMN, Lip JM, Noh JM, Normah A, Nabilah MN (2011). Identification and isolation of methyl gallate as a polar chemical marker for *Labisia pumila* Benth. J. Trop. Agric. Food. Sci..

[18] Tanaka T, Nonaka GI, Nishioka I (1983). 7-O-Galloyl-(+)-catechin and 3-O-galloylprocyanidin B-3 from *Sanguisorba officinalis*. Phytochemistry.

[19] Tanaka T, Nonaka GI, Nishioka I (1985). Punicafolin, an ellagitannin from the leaves of *Punica grantum*. Phytochemistry.

[20] Thuong PT, Kang JH, Na KM, Jin YW, Youn JU, Seong HY (2007). Anti-oxidant constituents from *Sedum takesimense*. Phytochemistry.

[21] Wang KJ, Yang CR, Zhang YJ (2007). Phenolic antioxidants from Chinese toon (fresh young leaves and shoots of *Toona sinensis*). Food Chem..

[22] Xin-Min C, Yoshida T, Hatano T, Fukushima M, Okuda T (1987). Galloylarbutin and other polyphenols from *Bergenia purpurascens*. Phytochemistry.

[23] Yang CM, Cheng HY, Lin TC, Chinag LC, Lin CC (2007). The in vitro activity of geraniin and 1,3,4,6-tetra-O-galloyl-β-D-flucose isolated from *Phyllanthus urinaria* against herpes simplex virus type 1 and type 2 infection. J. Ethnopharmacol..

[24] Yuan GQ, Li QQ, Qin J (2012). Isolation of methyl gallate from *Toxicodendron sylvestre* and its effect on tomato bacterial wilt. Plant Dis..

[25] Dickson RA, Houghton PJ, Hyhinds PJ, Gibbon S (2006). Antimicrobial, resistance-modifying effects, antioxidant and free radical scavenging activities of *Mezoneuron benthamianum* Bail., *Securinega virosa* Roxb. and Wlld. and *Microglossa pyrifolia* Lam. Phytother. Res..

[26] Wikler MA (2012). Methods for dilution antimicrobial susceptibility tests for bacteria that grow aerobically: approved standard. CLSI (NCCLS)..

[27] Norden CW, Wentzel H, Keleti E (1979). Comparison of techniques for measurement of in vitro antibiotic synergism. J. Infect. Dis..

[28] Yu HH, Kim KJ, Cha JD, Kim HK, Lee YE, Choi NY (2005). Antimicrobial activity of berberine alone and in combination with ampicillin or oxacillin against methicillin-resistant *Staphylococcus aureus*. J. Med. Food..

[29] Vu TT, Kim JC, Choi YH, Choi GJ, Jang KS, Choi TH (2013). Effect of gallotannins derived from *Sedum takesimense* on tomato bacterial wilt. Plant Dis..

[30] Odontuya G (2016). Anti-oxidative, acetylcholinesterase and pancreatic lipase inhibitory activities of compounds from *Dasiphora fruticosa Myricaria alopecuroides* and *Sedum hybridum*. Mong. J. Chem..

[31] Bensouici C, Kabouche A, Karioti A, Öztürk M, Duru ME, Bilia AR (2016). Compounds from *Sedum caeruleum* with antioxidant, anticholinesterase, and antibacterial activities. Pharm. Biol..

[32] Lee DS, Kang MS, Hwang HJ, Eom SH, Yang JY, Lee MS (2008). Synergistic effect between dieckol from *Ecklonia stolonifera* and β-lactams against methicillin-resistant *Staphylococcus aureus*. Biotechnol. Bioprocess. Eng..

[33] Lee DS, Eom SH, Kim YM, Kim HS, Yim MJ, Lee SH (2014). Antibacterial and synergic effects of gallic acid-*grafted*-chitosan with β-lactams against methicillin-resistant *Staphylococcus aureus* (MRSA). Can. J. Microbiol..

[34] Cantoni LAMJ, Wenger A (Glauser MP, Bille J). 1989. Comparative efficacy of amoxicillin-clavulanate, cloxacillin, and vancomycin against methicillin-sensitive and methicillin-resistant *Staphylococcus aureus* endocarditis in rats. J. Infect. Dis..

[35] Côté H, Pichette A, Simard F, Ouellette ME, Ripoll L, Mihoub M (2019). Balsacone C, a new antibiotic targeting bacterial cell membranes, inhibits clinical isolates of methicillin-resistant *Staphylococcus aureus* (MRSA) without inducing resistance. Front. Microbiol..

