# Supplementary material for: Synergistic Antibacterial Activity of an Active Compound Derived from Sedum takesimense against Methicillin-Resistant Staphylococcus aureus and Its Clinical Isolates
Source: J Microbiol Biotechnol. 2021 Jul 21;31(9):1288–94. doi: 10.4014/jmb.2105.05015 (PMC9705924; doi:10.4014/jmb.2105.05015)
Supplement: Supplementary file 1 [file jmb-31-9-1288-supple.pdf]

J. Microbiol. Biotechnol.
https://doi.org/10.4014/jmb.2105.05015

Table S1. The chemical shifts of hydroxyl proton in galloyl and glucose in the purified compound (TOGG; 1,2,4,6-tetra-O-galloyl-β-glucose).

**Position**	**Compounds**
Galloyl	
2’, 6’	7.10(2H, s)
2”, 6”	7.09(2H, s)
2’”, 6’”	7.05(2H, s)
2””, 6””	7.03(2H, s)
Glucose	
1	6.04(1H, d, 8.4)
2	5.35(1H, dd, 8.3, 9.6)
3	4.184.18(1H, t, 9.5)
4	5.37(1H, t, 9.7)
5	4.19(1H, m)
6	4.47(1H, dd, 2.2, 12.3)
	4.27(1H, dd, 2.2, 12.3)

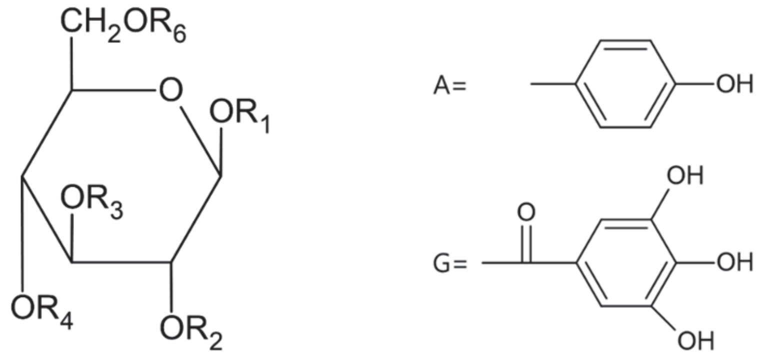

**Compound**	**R_1**	**R_2**	**R_3**	**R_4**	**R_6**
1,2,4,6-tetra-O-galloyl-β-glucose	G	G	H	G	G

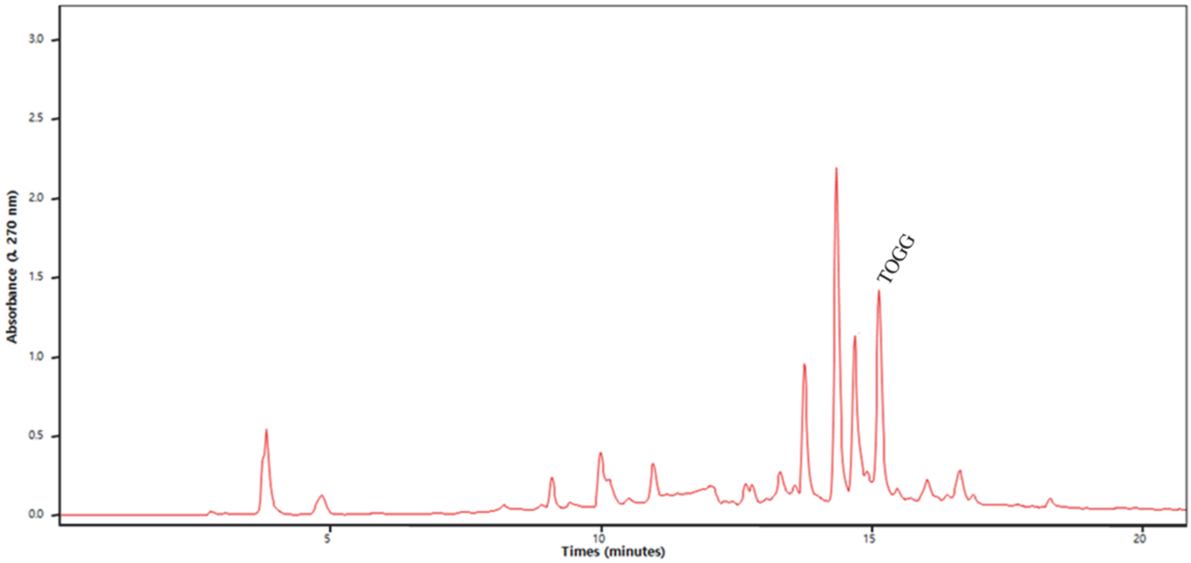

Fig. S1. HPLC chromatogram of TOGG derived from *S.takesimense*.

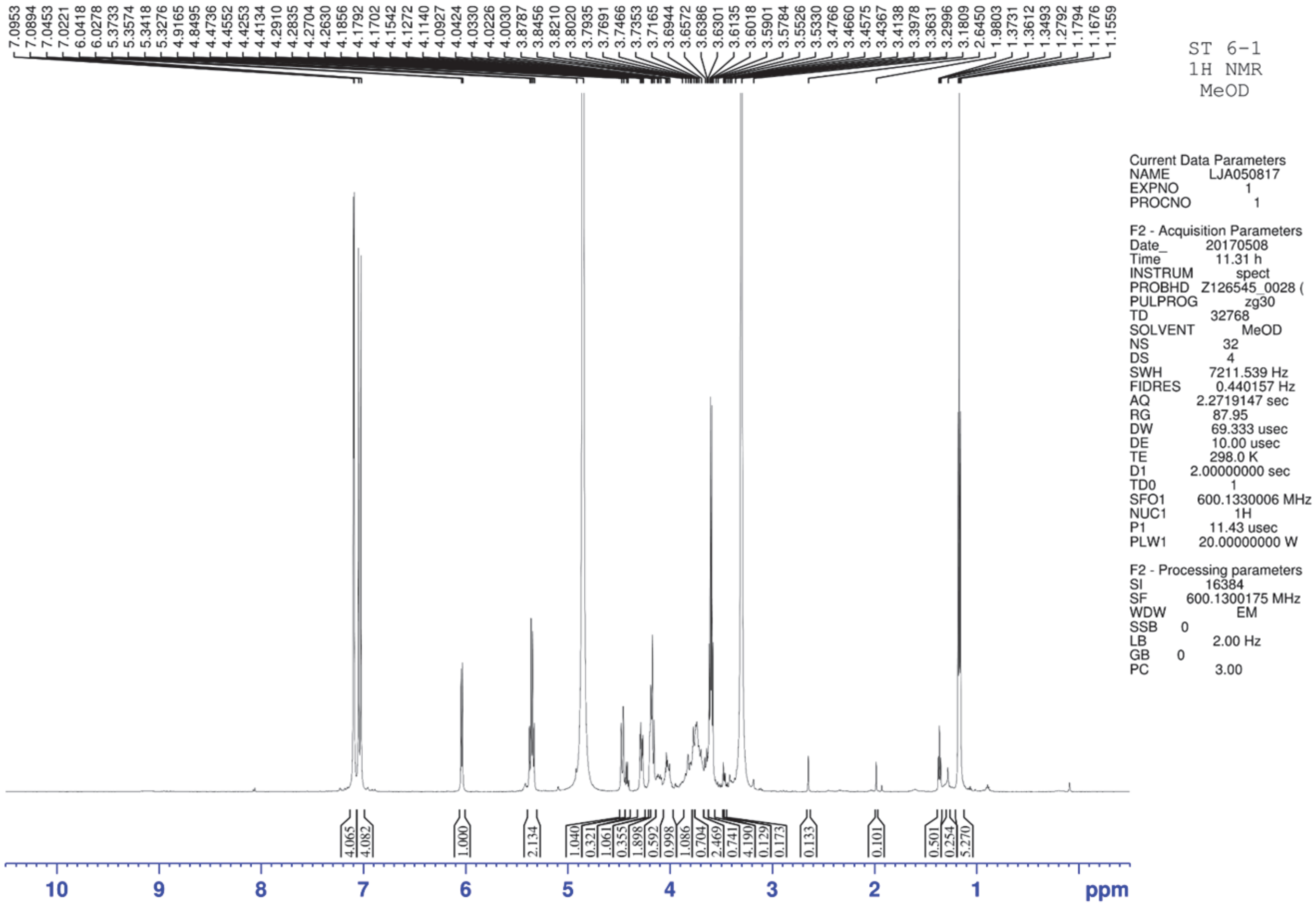

Fig. S2. ^{1}H-NMR spectra of the isolated and purified compound (TOGG; 1,2,4,6-tetra-O-galloyl-β-glucose).

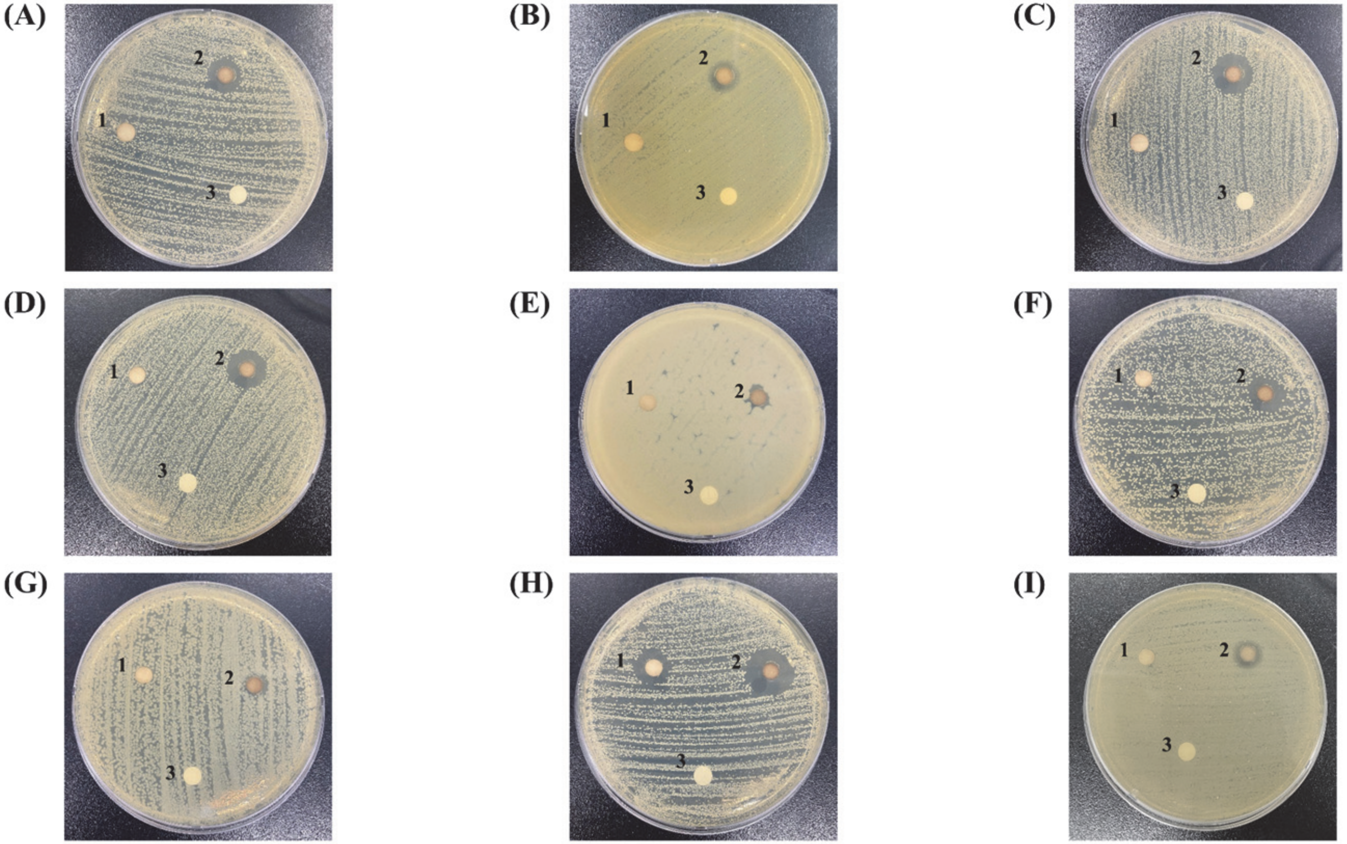

Fig S3. Results of the zone of inhibition assay using crude extract concentrations (1, 1mg/disc; 2, 5mg/disc, 3, distilled water) from *S. takesimense* **(A)**, *Escherichia coli*; **(B)**, *Pseudomonas aeruginosa*; **(C)**, *Salmonella typhimurium*; **(D)**, *Vibrio parahaemolyticus*; **(E)**, *Bacillus cereus*; **(F)**, *Listeria monocytogenes*; **(G)**, *Staphylococcus aureus*; **(H)**, *Staphylococcus epidermidis*; and **(I)**, *Cutibacterium acnes*.

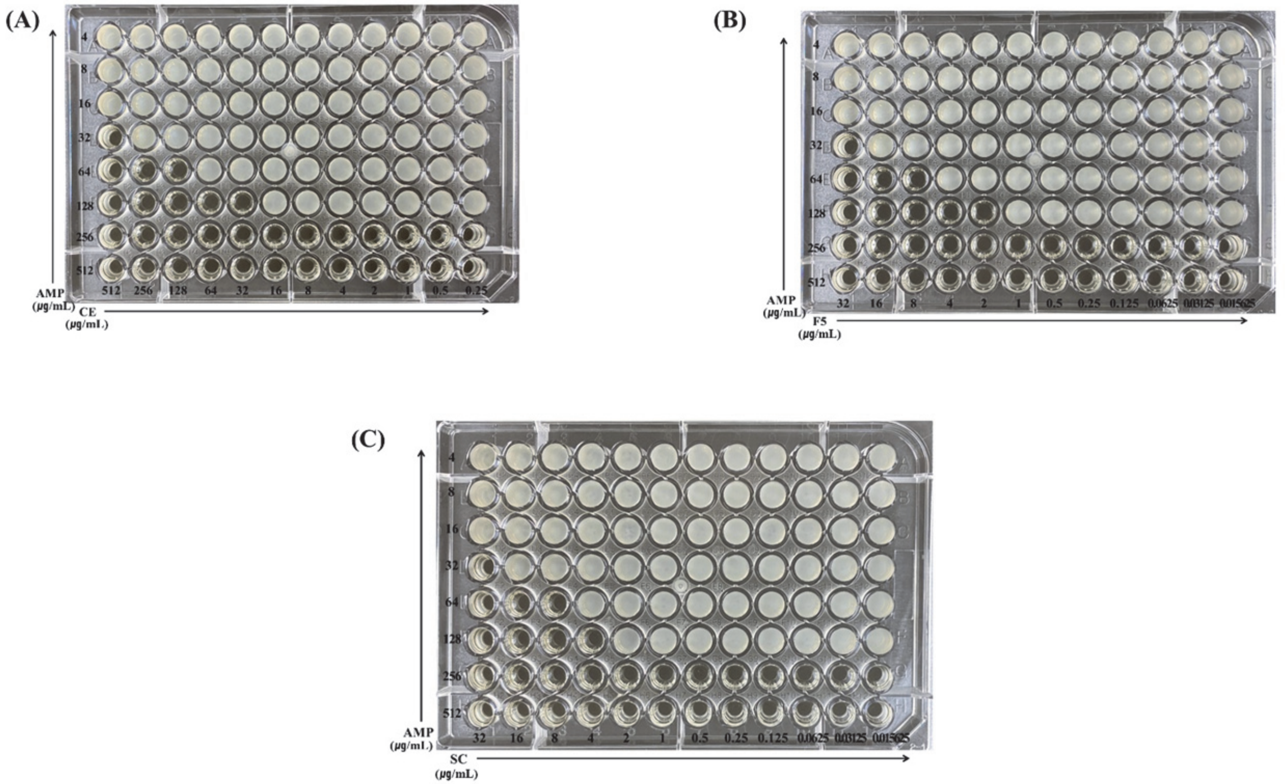

Fig. S4. Results of representative checkerboard assay (FICS, Fractional inhibitory concentration indexes) for the combination of crude extract (CE), fraction no. 5 (F5), and SC-single compound (TOGG) with ampicillin antibiotic against Methicillin-resistant *Staphylococcus aureus* KCCM 40511 (**A**, Crude extract combined with ampicillin; **B**, Fraction 5 combined with ampicillin; **C**, TOGG combined with ampicillin)
